# Robust Stabilization of Linear Time-Delay Systems under Denial-of-Service Attacks

**DOI:** 10.3390/s23135773

**Published:** 2023-06-21

**Authors:** Abdul-Wahid A. Saif, Sami El-Ferik, Siddig M. Elkhider

**Affiliations:** 1 Interdisciplinary Center of Smart Mobility and Logistics, King Fahd University of Petroleum and Minerals, P.O. Box 5067, Dhahran 31261, Saudi Arabia; 2 Control and Instrumentation Engineering Department, King Fahd University of Petroleum and Minerals, P.O. Box 5067, Dhahran 31261, Saudi Arabia

**Keywords:** time-delay systems, DoS attacks, PID-like state feedback control, robust control, LMI approach

## Abstract

This research examines new methods for stabilizing linear time-delay systems that are subject to denial-of-service (DoS) attacks. The study takes into account the different effects that a DoS attack can have on the system, specifically delay-independent and -dependent behaviour. The traditional proportional-integral-derivative (PID) acts on the error signal, which is the difference between the reference input and the measured output. The approach in this paper uses what we call the PID state feedback strategy, where the controller acts on the state signal. Our proposed strategy uses the Lyapunov–Krasovskii functional (LKF) to develop new linear matrix inequalities (LMIs). The study considers two scenarios where the time delay is either a continuous bounded function or a differentiable and time-varying function that falls within certain bounds. In both cases, new LMIs are derived to find the PID-like state feedback gains that will ensure robust stabilization. The findings are illustrated with numerical examples.

## 1. Introduction

Many researchers have been drawn to studying the stabilization and control of systems that are infinite-dimensional or have a time delay. Time delays are considered among the main causes of instability and poor performance in dynamic systems. A study of robust stability analysis and robust control design for time-delay systems (TDS) has been reported in [1].

The choice of an appropriate Lyapunov–Krasovskii functional (LKF) with additional parameters is important in developing sufficient stability conditions in the form of linear matrix inequalities (LMIs). The choice of LKF for linear state-delay systems is based on the type of the time-delay independent, applicable to delays of arbitrary size, or dependent, where the size of the delay is included.

State-derivative feedback methods have been used to design controllers for physical systems, such as mechanical systems [2], bridge systems with cable vibration [3], and car suspension systems [4,5,6,7], but the effect of the time delay was not taken into consideration in these applications. The time delay appears in many other physical systems such as water quality in streams [8], power systems [9], combustion in motor chambers [10], industrial-scale polymerization [11], and many others.

A denial-of-service (DoS) attack is a type of cyber-attack that aims to make a network resource unavailable to its intended users by overwhelming it with a flood of traffic or requests. This can cause delays in the network control system (NCS), as the system is unable to process legitimate requests due to the overwhelming amount of traffic. The delays can lead to slow or unresponsive network performance, which can impact the ability of the system to access the network and the services it provides. Additionally, the NCS may become overwhelmed, crash, or become unstable, causing further disruptions. Overall, a DoS attack on an NCS can have a significant impact on the availability and reliability of the system. The authors of [12] focused on NCSs that experience DoS attacks while transmitting signals from sensors to controllers through a communication network. The investigation specifically centred on stabilization control and stabilizing data-rate condition issues. The study considered a type of DoS attack that limits its frequency and duration. The authors of [13] addressed the stability issue of NCSs that are susceptible to DoS attacks. These attacks affect the transmission of control and measurement packets over communication networks, causing a time-varying delay in the system’s operation. The authors of [14] tackled the stability challenge of a specific category of NCSs that encounter network attacks. DoS attacks are comprehensively incorporated into the NCS’s modelling as a type of network attack on control systems. The study offers a closed-loop system with an error function for the NCSs in the presence of DoS attacks in the communication channel. DoS attacks are adequately taken into account in the system’s analysis. The authors of [15] focussed on the switching-like event-triggered control applied to NCSs that encounter malicious DoS attacks. To handle intermittent DoS attacks effectively and maintain the desired control performance, the study devised a switching-like event-triggered communication scheme (SETC). The system under investigation was transformed into a time-delay system. Then, with the constraint of the maximum number of allowable data dropouts caused by DoS attacks, the study derived a stability criterion and a stabilization criterion. The authors of [16] provided a solution to prevent DoS attacks and discrete events in a networked system. The system was modelled as an uncertain dynamic system with possible matched and mismatched perturbations and exogenous disturbances. To model the DoS attack duration and inter-event times, an approach accounting for the time delay of measurements between the sensor and controller over the network was proposed. As a result, an interval-time-delay system with uncertainties was formulated. To combat DoS attacks, a state–observer-based sliding mode controller was proposed, enabling the ideal sliding mode to be achieved. The authors of [17] focused on observer-based event-triggered control of a continuous linear system, subject to periodic DoS attacks, aiming to block data transmission in control channels. Initially, a resilient event-triggering scheme was proposed to counter any DoS attacks. Furthermore, an event-based switched system model was created to consider the effect of both the event-triggering scheme and DoS attacks together. The authors of [18] proposed a novel observer-based PID controller in order to ensure a desired security level and an upper bound on the quadratic cost criterion (QCC) for a kind of linear discrete time-delay system subject to cyber-attacks. These attacks (DoS and deception attacks) randomly occur according to two sequences of Bernoulli-distributed random variables with certain probabilities. Sufficient conditions were derived so that the closed-loop system was exponentially mean-square input-to-state stable, thereby achieving the desired security level. The authors of [19] discussed the resilient control of NCSs in the presence of DoS attacks, represented by a Markov process. To begin, the packet dropouts caused by DoS attacks were represented using a Markov process. Additionally, an NCS vulnerable to DoS attacks was modelled as a Markovian jump linear system. The Lyapunov theory was used to derive four theorems for the analysis of system stability and controller design. In order to reduce unnecessary signal transmissions between agents, an event-triggering scheme was employed along with a stochastic variable that follows a Bernoulli distribution to describe if communications among agents were affected by deception attack signals. The authors of [20] offered an asymmetric Lyapunov–Krasovskii functional (LKF) to address a leader-following consensus in multi-agent systems (MASs) subjected to deception attacks. The authors of [21,22] introduced a new strategy for controlling multiple unmanned aerial vehicles (UAVs) under cyber-attack using distributed data sharing (DDS) middleware. To protect UAVs, the connection between them and the overarching system, the DDS middleware was utilized, which made the system more resilient against these cyber-attacks. The authors of [23] presented a hybrid-triggered controller for stabilizing parabolic-type partial differential equations (PDEs) that experience deception attacks and disturbances. The study designed the hybrid-triggered controller by merging time- and event-triggered controllers, aided by a Bernoulli random variable. A non-linear function was used to represent the deception attack signal, with the probability of attack occurrence determined by the Bernoulli random variable. To mitigate the effects of disturbances, an $H_{\infty }$ performance was employed. The study applied a Lyapunov–Krasovskii functional (LKF) to analyse the stabilization of the selected PDE under the proposed controller, with the stabilization conditions derived in terms of linear matrix inequalities (LMIs).

This paper will examine the advancement of robust stabilization techniques for systems that are vulnerable to DoS attacks. Specifically, it will address the issue of robust stabilization through the use of a PID-like state feedback controller for DoS attacks that result in both delay-independent and -dependent effects. A new approach for obtaining PID-like parameters will be presented, involving formulating and solving LMIs based on the appropriate choice of the Lyapunov–Krasovskii functional (LKF). The time delay caused by DoS attacks is assumed to be continuous and bounded for delay-independent cases, and differentiable and time-varying with upper-bound relations for delay-dependent cases. A numerical example will be provided to demonstrate the theoretical developments proposed.

The paper is organized as follows. Section 2 presents some preliminary results with the formulation of the PID-like state feedback controller. The closed-loop system is formulated. The main results of this work are stated in Section 3 and its subsections. Section 4 presents the simulation results, while the conclusion and future work are given in Section 5.

**Notations:**
  $W^{t},\;W^{-1}$ and $∥W∥$ denote the transpose, inverse, and induced norm of any square matrix *W*, respectively. W>0 (W<0) stands for an asymmetrical and positive (negative) definite matrix *W*. The *n*-dimensional Euclid *n* space is denoted by $R^{n}$ and *I* stands for the identity matrix with appropriate dimension. The symbol ∗ is used in some matrix expressions to induce a symmetrical structure.

## 2. Problem Definition

The following class of linear time-delay (LTD) systems subjected to DoS attacks will be considered:

(1) $$\begin{array}{ccc} \dot{x}\left(t\right) & = & A_{o}x\left(t\right)+A_{do}x\left(t-\tau \left(t\right)\right)+B_{o}u\left(t\right)+\Gamma_{o}w\left(t\right)\\ x(\phi ) & = & w(\phi ),\phi \in [-\tau ,0]\\ z(t) & = & G_{o}x\left(t\right)+G_{do}x\left(t-\tau \left(t\right)\right)+\Phi_{o}w\left(t\right) \end{array}$$

where $x\left(t\right)\in {ℜ}^{n},$$u\left(t\right)\in {ℜ}^{p}$, $z\left(t\right)\in {ℜ}^{q}$ and $w\left(t\right)\in {ℜ}^{q}$ are the state vector, control input, observed output and disturbance input, respectively. It is assumed that the disturbance w(t) belongs to $L_{2}\left[0,\infty \right)$. τ(t)>0 is a time delay caused by DoS attacks. w(ϕ) is the initial condition which is assumed to be differentiable in [−τ,0]. $A_{0}\in {ℜ}^{nxn},B_{0}\in {ℜ}^{nxp},G_{o}\in {ℜ}^{qxn},G_{d0}\in {ℜ}^{qxn},A_{do}\in {ℜ}^{nxn}$ and $\Gamma_{o}\in {ℜ}^{nxq},\Phi_{o}\in {ℜ}^{qxq}$ are real and known constant matrices.

The problem that will be solved is to find the PID-like constant gains $K_{D}\in {ℜ}^{pxn},K_{I}\in {ℜ}^{pxn},K_{P}\in {ℜ}^{pxn}$ such that the following conditions hold:

(1) Matrix $\left(I-B_{0}K_{D}\right)$ has full rank.
(2) The following PID-like state feedback controller is proposed
  (2) $$u\left(t\right)=K_{P}x\left(t\right)+K_{D}\dot{x}\left(t\right)+K_{I}\int_{t-\rho }^{t}x\left(s\right)ds$$
  where ρ is the upper bound on the time delay produced by DoS attacks, $K_{p}$ is a proportional gain designed to ensure internal stability, and $K_{D}$ and $K_{I}$ are to meet the control objectives.

Then, from (1) and (2), the closed-loop system is written as

(3) $$\begin{array}{ccc} \left(I-B_{o}K_{D}\right)\dot{x}\left(t\right) & = & \left(A_{o}+B_{o}K_{P}\right)x\left(t\right)+A_{do}x\left(t-\tau \left(t\right)\right)\\ & & +B_{o}K_{I}\int_{t-\rho }^{t}x\left(s\right)ds+\Gamma_{o}w\left(t\right)\\ & = & A_{s}x\left(t\right)+A_{do}x\left(t-\tau \left(t\right)\right)\\ & & +B_{o}K_{I}\int_{t-\rho }^{t}x\left(s\right)ds+\Gamma_{o}w\left(t\right)\\ z(t) & = & G_{o}x\left(t\right)+G_{do}x\left(t-\tau \left(t\right)\right)\\ & + & \Phi_{o}w\left(t\right),\;\\ A_{s} & = & A_{o}+B_{o}K_{P} \end{array}$$

Assuming that $\left[I-B_{o}K_{D}\right]$ is well defined, the closed-loop system (4) has the following form.

(4) $$\begin{array}{ccc} \dot{x}\left(t\right) & = & {\left(I-B_{o}K_{D}\right)}^{-1}\\ & * & \left\{\begin{array}{c} A_{s}x\left(t\right)+A_{do}x\left(t-\tau \left(t\right)\right)\\ +B_{o}K_{I}\int_{t-\rho }^{t}x\left(s\right)ds+\Gamma_{o}w\left(t\right) \end{array}\right\}\\ & = & G_{o}x\left(t\right)+G_{do}x\left(t-\tau \left(t\right)\right)+\Phi_{o}w\left(t\right) \end{array}$$

The following changes of variables are introduced to deal with the system given in (4)

(5) $$\upsilon \left(t\right)=\int_{t-\rho }^{t}x\left(s\right)ds$$

Then

(6) $$\dot{\upsilon }\left(t\right)=x\left(t\right)-x\left(t-\rho \right)$$

Appending (6) to system (4) and define $\zeta \left(t\right)=:{\left[\begin{array}{cc} x^{t}\left(t\right) & \upsilon^{t}\left(t\right) \end{array}\right]}^{t}$, the following augmented system, involving two time-delay variables produced by DoS attacks is obtained

(7) $$\begin{array}{ccc} \overset{\cdot }{\zeta }\left(t\right) & = & A_{a}\zeta \left(t\right)+A_{c}\zeta \left(t-\rho \right)+A_{do}\zeta \left(t-\tau \left(t\right)\right)\\ & & +{\hat{\Gamma }}_{o}w\left(t\right) \end{array}$$

(8) $$\begin{array}{ccc} z(t) & = & {\hat{G}}_{o}\zeta \left(t\right)+{\hat{G}}_{do}\zeta \left(t-\tau \left(t\right)\right)+\Phi_{o}w\left(t\right) \end{array}$$

where

$$\begin{array}{ccc} A_{a} & = & \left[\begin{array}{cc} {\left(I-B_{o}K_{D}\right)}^{-1}A_{s} & {\left(I-B_{o}K_{D}\right)}^{-1}B_{o}K_{I}\\ I & 0 \end{array}\right]\\ A_{c} & = & \left[\begin{array}{cc} 0 & 0\\ -I & 0 \end{array}\right]\\ A_{do} & = & \left[\begin{array}{cc} {\left(I-B_{o}K_{D}\right)}^{-1}A_{do} & 0\\ 0 & 0 \end{array}\right]\\ {\hat{\Gamma }}_{o} & = & \left[\begin{array}{c} {\left(I-B_{o}K_{D}\right)}^{-1}\Gamma_{o}\\ 0 \end{array}\right]\\ {\hat{G}}_{o} & = & \left[\begin{array}{cc} G_{o} & 0 \end{array}\right]\\ {\hat{G}}_{do} & = & \left[\begin{array}{cc} G_{do} & 0 \end{array}\right] \end{array}$$

In the following, the controller gains $K_{P},\;K_{D}$ and $K_{I}$ will be determined for two DoS attack behaviours:

Case 1 (Delay-independent): The time delay caused by DoS attack τ(t) is continuous and satisfies
  0≤τ(t)≤ρ,∀t≥0
Case 2 (Delay-dependent): The time delay caused by DoS attack τ(t) is continuous, differentiable and satisfies
  $$0\leq \tau \left(t\right)\leq \rho ,\;\dot{\tau }\left(t\right)\leq \mu$$
  where the bounds ρ and μ are known. From ref. [11], the usual bounding relation μ<1, but in this work it is expanded to μ<3. This new upper bound on μ is shown later in the proof of Theorem 2, contributing to others’ work.

## 3. Ltd under DoS Attack Control Design

### 3.1. DoS Attacks Causing Unknown Time-Delay Design

Let the time delay produced by DoS attack τ(t) be continuous and unknown, as described in Case 1. The next theorem identifies a delay-independent LMI-based condition for PID-like state feedback stabilization of system (1) with $H_{\infty }$ performance bound γ to overcome the effectiveness of the DoS attacks.

**Theorem** **1.** *For the DoS behaviour defined in Case 1, system (1) is delay-independent and asymptotically stable with performance bound γ under a PID-like state feedback controller*

(9) $$u\left(t\right)=K_{P}x\left(t\right)+K_{D}\dot{x}\left(t\right)+K_{I}\int_{t-\rho }^{t}x\left(s\right)ds$$

*if there exist positive definite matrices $X_{x},$$Z_{x},Q_{x},N_{0},$ and $K_{I},H,L_{x},R_{x},Y,W$ such that the following LMI*

(10) $$\left[\begin{array}{cc} E_{11} & E_{12}\\ E_{21} & E_{22} \end{array}\right]<0$$

*has a feasible solution, where*

$$\begin{array}{ccc} E_{11} & = & \left[\begin{array}{ccc} \Pi_{11}+G_{o}^{t}G_{o} & \Pi_{13} & A_{do}+G_{o}^{t}G_{do}\\ {}* & -N_{0}+2I & 0\\ {}* & * & -Z_{x}+G_{do}^{t}G_{do} \end{array}\right]\\ E_{12} & = & \left[\begin{array}{cccccc} 0 & 0 & 0 & \Gamma_{0}+G_{o}^{t}\Phi_{o} & R_{x} & L_{x}\\ 0 & -I & 0 & 0 & 0 & 0\\ 0 & 0 & 0 & G_{do}^{t}\Phi_{o} & 0 & 0 \end{array}\right]\\ E_{21} & = & E_{12}^{T}\\ E_{22} & = & \left[\begin{array}{cccccc} -I & 0 & 0 & 0 & 0 & 0\\ {}* & -Q_{x} & 0 & 0 & 0 & 0\\ {}* & * & -I & 0 & 0 & 0\\ {}* & * & * & -\Pi_{24} & 0 & 0\\ {}* & * & * & * & -Z_{x} & 0\\ {}* & * & * & * & * & -Q_{x} \end{array}\right] \end{array}$$

$$\begin{array}{ccc} \Pi_{11} & = & A_{0}X_{x}+X_{x}A_{0}^{t}+B_{0}W+W^{t}B_{0}^{t}\\ & + & A_{0}Y^{t}B_{0}^{t}+B_{0}YA_{0}^{t}+H+H^{t}\\ \Pi_{13} & = & B_{0}K_{I}+X_{x}+B_{0}Y\\ \Pi_{24} & = & \gamma^{2}I-\Phi_{o}^{t}\Phi_{o} \end{array}$$

*Moreover, the feedback gains are given by $K_{P}={\mathrm{WX}}_{x}^{-1},$$K_{D}=-{\mathrm{YX}}_{x}^{-1}$ and $K_{I}$ is computed directly from element (1,2) of $E_{11}$.*

**Proof.**  First, the asymptotic stability of the closed-loop system in (7) is stabilized when ω(.)≡0, i.e.,

(11) $$\overset{\cdot }{\zeta }\left(t\right)=A_{a}\zeta \left(t\right)+A_{c}\zeta \left(t-\rho \right)+A_{do}\zeta \left(t-\tau \right)$$

Define the selective LKF

(12) $$V_{1}\left(t\right)=\zeta {\left(t\right)}^{t}P\zeta \left(t\right)+\int_{t-\tau }^{t}\zeta^{t}\left(s\right)Z\zeta \left(s\right)ds+\int_{t-\rho }^{t}\zeta^{t}\left(s\right)Q\zeta \left(s\right)ds$$

where

$$P=\left[\begin{array}{cc} P_{x} & 0\\ 0 & I \end{array}\right],\;Z=\left[\begin{array}{cc} Z_{x} & 0\\ 0 & I \end{array}\right],\;\;Q=\left[\begin{array}{cc} Q_{x} & 0\\ 0 & I \end{array}\right]$$

are positive definite unknown matrices. Differentiating $V_{1}\left(t\right)$ along the solutions of (11) and with some algebraic manipulation, we obtain

(13) $${\overset{\cdot }{V}}_{1}\left(t\right)=\eta^{t}\left(t\right)\Omega \eta \left(t\right)$$

where

$$\begin{array}{ccc} \Omega & = & \left[\begin{array}{ccc} PA_{a}+A_{a}P+Z+Q & PA_{do} & PA_{c}\\ {}* & -Z & 0\\ {}* & * & -Q \end{array}\right]<0\\ \eta (t) & = & {\left[\begin{array}{ccc} \zeta {\left(t\right)}^{t} & \zeta {\left(t-\tau \right)}^{t} & \zeta {\left(t-\rho \right)}^{t} \end{array}\right]}^{t} \end{array}$$

Ω<0 implies that $\overset{\cdot }{V}\left(t\right)<0.$ Using the congruent transformation $diag\left[\begin{array}{ccc} T_{1} & I & I \end{array}\right]$ to Ω with $Y=-K_{D}X_{x},$$W=K_{P}X_{x},$ and

$$\begin{array}{ccc} T_{1} & = & \left[\begin{array}{cc} \left(I-B_{0}K_{D}\right)P_{x}^{-1} & 0\\ 0 & I \end{array}\right],\\ P^{-1} & : & =X=\left[\begin{array}{cc} X_{x} & 0\\ 0 & I \end{array}\right] \end{array}$$

then making use of the 𝒮-procedure [12] with some algebraic manipulations, after expanding and simplifying its elements in (12) Ω in (13) becomes

(14) $$\begin{array}{ccc} \Omega & = & \\ & & \;\;\;\left[\begin{array}{cccccc} \Pi_{11}+\Pi_{12} & \Pi_{13} & A_{do} & 0 & 0 & 0\\ {}* & -N_{0}+2I & 0 & 0 & -I & 0\\ {}* & * & -Z_{x} & 0 & 0 & 0\\ {}* & * & * & -I & 0 & 0\\ {}* & * & * & * & -Q_{x} & 0\\ {}* & * & * & * & * & -I \end{array}\right]\;\\ & < & 0 \end{array}$$

where

$$\begin{array}{ccc} \Pi_{12} & = & \left(X_{x}+B_{0}Y\right)Z_{x}{\left(X_{x}+B_{0}Y\right)}^{t}\\ & & +\left(X_{x}+B_{0}Y\right)Q_{x}{\left(X_{x}+B_{0}Y\right)}^{t}\\ & = & \left(X_{x}+B_{0}Y\right)Z_{x}Z_{x}^{-1}Z_{x}{\left(X_{x}+B_{0}Y\right)}^{t}\\ & & +\left(X_{x}+B_{0}Y\right)Q_{x}Q_{x}^{-1}Q_{x}{\left(X_{x}+B_{0}Y\right)}^{t}\\ & = & R_{x}Z_{x}^{-1}R_{x}^{t}+L_{x}Q_{x}^{-1}L_{x}^{t}\\ H & = & B_{0}K_{P}X_{x}K_{D}^{t}B_{0}^{t},\;\;\;R_{x}=\left(X_{x}+B_{0}Y\right)Z_{x},\;\;\;\\ L_{x} & = & \left(X_{x}+B_{0}Y\right)Q_{x}\\ R_{x} & = & \left(X_{x}+B_{0}Y\right)Z_{x} \end{array}$$

Using Schur’s complement on $\Pi_{12}$ in (14), the asymptotic stability of system (4) follows from (10) since ${\overset{\cdot }{V}}_{1}\left(t\right)<0$.

Inequality (14) is bi-linear in $X_{x},Y$, $Z_{x}$ and $Q_{x}$. Next, it is converted into an LMI to be able to obtain the controller gains. To do this, the performance measure is included as follows. Let the performance measure *J* be defined as

(15) $$J=\int_{0}^{\infty }\left[z{\left(s\right)}^{t}z\left(s\right)-\gamma^{2}w^{t}\left(s\right)w\left(s\right)\right]ds$$

Assume $w\left(t\right)\in L_{2}\left(0,\infty \right)\neq 0$ and the initial condition x(0)=0, we thus have

(16) $$\begin{array}{ccc} \;J & = & \int_{0}^{\infty }\left[z{\left(s\right)}^{t}z\left(s\right)-\gamma^{2}w^{t}\left(s\right)w\left(s\right)+{\left.{\overset{\cdot }{V}}_{1}\left(t\right)\right|}_{\left(7\right)}\right]ds\\ & & -{\left.{\overset{\cdot }{V}}_{1}\left(t\right)\right|}_{\left(7\right)}\\ & \leq & \int_{0}^{\infty }\left[z{\left(s\right)}^{t}z\left(s\right)-\gamma^{2}w^{t}\left(s\right)w\left(s\right)+{\left.{\overset{\cdot }{V}}_{1}\left(t\right)\right|}_{\left(7\right)}\right]ds \end{array}$$

First, from (16), we evaluate

(17) $$z^{t}\left(s\right)z\left(s\right)-\gamma^{2}\omega^{t}\left(s\right)\omega \left(s\right)={\bar{\eta }}^{t}\left(t\right)\Sigma_{\infty }\bar{\eta }\left(t\right)$$

where

$$\bar{\eta }\left(t\right)={\left[\begin{array}{cccc} \zeta {\left(t\right)}^{t} & \zeta {\left(t-\tau \right)}^{t} & \zeta {\left(t-\rho \right)}^{t} & \omega^{t}\left(t\right) \end{array}\right]}^{t}$$

(18) $$\Sigma_{\infty }=\left[\begin{array}{cccc} {\hat{G}}_{o}^{t}{\hat{G}}_{o} & {\hat{G}}_{o}^{t}{\hat{G}}_{do} & 0\; & {\hat{G}}_{o}^{t}\Phi_{o}\\ {\hat{G}}_{do}^{t}{\hat{G}}_{o} & {\hat{G}}_{do}^{t}{\hat{G}}_{do} & 0 & {\hat{G}}_{do}^{t}\Phi_{o}\\ 0 & 0 & 0 & 0\\ \Phi_{o}^{t}{\hat{G}}_{o} & \Phi_{o}^{t}{\hat{G}}_{do} & 0 & -\gamma^{2}I+\Phi_{o}^{t}\Phi_{o} \end{array}\right]$$

$$\begin{array}{ccc} {\hat{G}}_{o}^{t}{\hat{G}}_{o} & = & \left[\begin{array}{cc} G_{o}^{t}G_{o} & 0\\ 0 & 0 \end{array}\right]\\ {\hat{G}}_{o}^{t}{\hat{G}}_{do} & = & \left[\begin{array}{cc} G_{o}^{t}G_{do} & 0\\ 0 & 0 \end{array}\right]\\ {\hat{G}}_{o}^{t}\Phi_{o} & = & \left[\begin{array}{c} G_{o}^{t}\Phi_{o}\\ 0 \end{array}\right]\\ {\hat{G}}_{do}^{t}\Phi_{o} & = & \left[\begin{array}{c} G_{do}^{t}\Phi_{o}\\ 0 \end{array}\right]\\ {\hat{G}}_{do}^{t}{\hat{G}}_{do} & = & \left[\begin{array}{cc} G_{do}^{t}G_{do} & 0\\ 0 & 0 \end{array}\right] \end{array}$$

Next, (13) is modified by adding the coefficient of w(s) from (7). In terms of $\bar{\eta }$, it becomes as

$${\overset{\cdot }{V}}_{1}\left(t\right)={\bar{\eta }}^{t}\left(t\right)\bar{\Omega }\bar{\eta }\left(t\right)$$

where

(19) $$\begin{array}{ccc} \bar{\Omega } & = & \\ & & \;\left[\begin{array}{cccc} PA_{a}+A_{a}P+Z+Q & PA_{do} & PA_{c} & P{\hat{\Gamma }}_{0}\\ {}* & -Z & 0 & 0\\ {}* & * & -Q & 0\\ {}* & * & * & 0 \end{array}\right] \end{array}$$

Applying the congruent transformation $diag\left[\begin{array}{cccc} T_{1} & I & I & I \end{array}\right]$ on $\bar{\Omega },$ and making use of the 𝒮-procedure [12] with some algebraic manipulations, (19) becomes

(20) $$\begin{array}{ccc} \Xi_{t} & = & \left[\begin{array}{cc} \Pi_{11}+\Pi_{12} & B_{0}K_{I}+X_{x}+B_{0}Y\\ {}* & -N_{0}+2I\\ {}* & *\\ {}* & *\\ {}* & *\\ {}* & *\\ {}* & * \end{array}\right.\\ & & \left.\begin{array}{ccccc} A_{do} & 0 & 0 & 0 & \Gamma_{0}\\ 0 & 0 & -I & 0 & 0\\ -Z_{x} & 0 & 0 & 0 & 0\\ {}* & -I & 0 & 0 & 0\\ {}* & * & -Q_{x} & 0 & 0\\ {}* & * & * & -I & 0\\ {}* & * & * & * & 0 \end{array}\right] \end{array}$$

Now, expanding (18) and combining it with (20) to obtain

$$z^{t}\left(s\right)z\left(s\right)-\gamma^{2}\omega^{t}\left(s\right)\omega \left(s\right)+\overset{\cdot }{V_{1}}\left(s\right)=\tilde{\eta }\left(s\right)\Xi_{s}\tilde{\eta }\left(s\right)$$

where

$$\begin{array}{ccc} \Xi_{s} & = & \left[\begin{array}{cc} \Xi_{s11} & \Xi_{s12}\\ \Xi_{s21} & \Xi_{s22} \end{array}\right]\\ \Xi_{s11} & = & \left[\begin{array}{cc} \Pi_{11}+\Pi_{12}+G_{o}^{t}G_{o} & B_{0}K_{I}+X_{x}+B_{0}Y\\ {}* & -N_{0}+2I \end{array}\right]\\ \Xi_{s12} & = & \left[\begin{array}{ccccc} A_{do}+G_{o}^{t}G_{do} & 0 & 0 & 0 & \Gamma_{0}+G_{o}^{t}\Phi_{o}\\ 0 & 0 & -I & 0 & 0\\ -Z_{x}+G_{do}^{t}G_{do} & 0 & 0 & 0 & G_{do}^{t}\Phi_{o} \end{array}\right]\\ \Xi_{s21} & = & \Xi_{s12}^{T}\\ \Xi_{s22} & = & \\ & & \;\left[\begin{array}{ccccc} -Z_{x}+G_{do}^{t}G_{do} & 0 & 0 & 0 & G_{do}^{t}\Phi_{o}\\ {}* & -I & 0 & 0 & 0\\ {}* & * & -Q_{x} & 0 & 0\\ {}* & * & * & -I & 0\\ {}* & * & * & * & -\gamma^{2}I+\Phi_{o}^{t}\Phi_{o} \end{array}\right] \end{array}$$

Using Schur’s complement on $\Pi_{12}$, (10) is obtained with the controller gains. Since $\Xi_{s}<0,$ it follows that J<0 and ${∥z(t)∥}_{2}<\gamma {∥w(t)∥}_{2}$, and the proof of the $H_{\infty }$ performance bound is achieved. □

**Remarks:**
  - The solution to inequality (10) will result in a sub-optimal one. The optimal gains of the delay-independent asymptotically stabilized controller can be determined by solving the following convex minimization problem
    (21) $$\underset{\begin{array}{c} wrt\;X_{x}\;>\;0,\;Z_{x}\;>\;0,\;Q_{x}\;>\;0,\;N_{0}\;>\;0,\;L_{x},\;R_{x}\;K_{I},\;H,\;Y,\;W\\ \mathrm{subjet}\;\mathrm{to}\;\mathrm{LMI}\;(10) \end{array}}{\mathrm{Minimize}\;\gamma }$$
  - The conventional state feedback stabilization controller
    $$u\left(t\right)=K_{P}x\left(t\right)$$
    is obtained as stated by the next lemma.

**Lemma** **1.** *For the DoS behaviour defined in Case 1, system (1) with state feedback $u\left(t\right)=K_{P}x\left(t\right)$ is delay-independent and asymptotically stabilized with $H_{\infty }$ performance bound γ if there exist matrices $X_{x}>0,Z_{x}>0,Q_{x}>0,N_{0}>0$ and W such that the following LMI*

(22) $$\begin{array}{ccc} 𝒮 & = & \left[\begin{array}{cc} {𝒮}_{1} & {𝒮}_{2} \end{array}\right]<0\\ {𝒮}_{1} & = & \left[\begin{array}{ccc} {\bar{\Pi }}_{11}+G_{o}^{t}G_{o} & X_{x} & A_{do}+G_{o}^{t}G_{do}\\ {}* & -N_{0}+2I & 0\\ {}* & * & -Z_{x}+G_{do}^{t}G_{do}\\ {}* & * & *\\ {}* & * & *\\ {}* & * & *\\ {}* & * & * \end{array}\right]\\ {𝒮}_{2} & = & \left[\begin{array}{cccc} 0 & 0 & 0 & \Gamma_{0}+G_{o}^{t}\Phi_{o}\\ 0 & -I & 0 & 0\\ 0 & 0 & 0 & G_{do}^{t}\Phi_{o}\\ -I & 0 & 0 & 0\\ {}* & -Q_{x} & 0 & 0\\ {}* & * & -I & 0\\ {}* & * & 0 & -\gamma^{2}I+\Phi_{o}^{t}\Phi_{o} \end{array}\right] \end{array}$$

*has a feasible solution, where*

$${\bar{\Pi }}_{11}=A_{0}X_{x}+X_{x}A_{0}^{t}+B_{0}W+W^{t}B_{0}^{t}$$

*Moreover, the feedback gain is given by $K_{P}={\mathrm{WX}}_{x}^{-1}.$*

**Proof.**  The proof of this lemma is obtained by setting $K_{I}=H=L_{x}=R_{x}=Y=0$ in Theorem 1. □

**Remark** **1.** *The following linear-controlled delay-less system is*

(23) $$\begin{array}{ccc} \dot{x}\left(t\right) & = & {\left(I-B_{o}K_{D}\right)}^{-1}\left\{A_{s}x\left(t\right)+\Gamma_{o}w\left(t\right)\right\}\\ z(t) & = & G_{o}x\left(t\right)+\Phi_{o}w\left(t\right) \end{array}$$

*is obtained by setting $A_{do}=0,G_{do}=0,$ and $K_{I}=0$ in Theorem 1. This special result is stated in the following corollary.*

**Corollary** **1.** *System (23) with PD-like state feedback control $u\left(t\right)=K_{P}x\left(t\right)+K_{D}\dot{x}\left(t\right)$ is asymptotically stabilized with $H_{\infty }$ performance bound γ>0 if there exist matrices $X_{x}>0,Z_{x}>0,Q_{x}>0,N_{0}>0,L_{x},R_{x},Y,$ and W such that the following LMI*

(24) $$G=\left[\begin{array}{cc} G_{1} & G_{2} \end{array}\right]<0$$

$$\begin{array}{ccc} G_{1}\; & = & \left[\begin{array}{cccc} \Pi_{11}+G_{o}^{t}G_{o} & X_{x}+B_{0}Y & 0 & 0\\ {}* & -N_{0}+2I & 0 & 0\\ {}* & * & -Z_{x} & 0\\ {}* & * & * & -I\\ {}* & * & * & *\\ {}* & * & * & *\\ {}* & * & * & *\\ {}* & * & * & *\\ {}* & * & * & * \end{array}\right]\\ G_{2} & = & \left[\begin{array}{ccccc} 0 & 0 & \Gamma_{0}+G_{o}^{t}\Phi_{o} & R_{x} & L_{x}\\ -I & 0 & 0 & 0 & 0\\ 0 & 0 & 0 & 0 & 0\\ 0 & 0 & 0 & 0 & 0\\ -Q_{x} & 0 & 0 & 0 & 0\\ {}* & -I & 0 & 0 & 0\\ {}* & * & -\gamma^{2}I+\Phi_{o}^{t}\Phi_{o} & 0 & 0\\ {}* & * & * & -Z_{x} & 0\\ {}* & * & * & * & -Q_{x} \end{array}\right] \end{array}$$

*has a feasible solution. Moreover, the feedback gains of the PD controller are given by $K_{D}=-{\mathrm{YX}}_{x}^{-1},K_{P}={\mathrm{WX}}^{-1}.$*

### 3.2. DoS Attacks Causing Time-Varying Delay Design

In this section, we will address the DoS behaviour described in Case 2, i.e., τ(t) is continuous and differentiable. The results presented will be in the form of new LMI characterization for delay-dependent stabilization by the PID-like state feedback controller. The following Leibniz–Newton formula will be used

(25) $$\zeta \left(t-\tau \left(t\right)\right)=\zeta \left(t\right)-\int_{t-\tau (t)}^{t}\dot{\zeta }\left(s\right)ds$$

Considering the transformed closed-loop systems (7) and (8), the following theorem is established:

**Theorem** **2.** *Consider the DoS behaviour defined in Case 2. The systems (7) and (8) are delay-dependent and asymptotically stabilized with $H_{\infty }$ performance bound γ if there exist positive definite matrices $X_{x},$$Z_{x},Q_{x},H,$$M_{x},{\left\{N_{i}>0\right\}}_{i=0}^{4},L_{x},R_{x},$${𝒮}_{x},\Theta_{x},{\tilde{\Phi }}_{x},\Psi_{x}$ and $K_{I},W,Y$ such that the following LMI*

(26) $$\tilde{H}=\left[\begin{array}{ccc} H_{11} & H_{12} & H_{13}\\ {}* & H_{22} & H_{23}\\ {}* & * & H_{33} \end{array}\right]<0$$

*has a feasible solution for all 0≤τ(t)≤ρ,$\dot{\tau }\left(t\right)\leq \mu ,$ where*

$$\begin{array}{ccc} H_{11} & = & \left[\begin{array}{ccc} \Omega_{11}+{\hat{G}}_{o}^{t}{\hat{G}}_{o} & \Omega_{12}+{\hat{G}}_{o}^{t}{\hat{G}}_{do} & \Omega_{13}\\ • & -\Omega_{22}+{\hat{G}}_{do}^{t}{\hat{G}}_{do} & 0\\ • & • & -\Omega_{33} \end{array}\right]\\ H_{12} & = & \left[\begin{array}{ccc} \Omega_{14} & {\hat{\Gamma }}_{1}+{\hat{G}}_{o}^{t}\Phi_{o} & \rho \Omega_{15}\\ -\rho \;\Omega_{24} & {\hat{G}}_{do}^{t}\Phi_{o} & \rho \;\Omega_{25}\\ 0 & 0 & \rho \Omega_{35} \end{array}\right]\\ H_{13} & = & \left[\begin{array}{cccc} \Omega_{16} & \Omega_{17} & \Omega_{18} & \Omega_{18}\\ 0 & 0 & 0 & 0\\ 0 & 0 & 0 & 0 \end{array}\right] \end{array}$$

$$\begin{array}{ccc} H_{22} & = & \left[\begin{array}{ccc} -\rho \Omega_{44} & 0 & 0\\ • & -\gamma^{2}I+\Phi_{o}^{t}\Phi_{o} & 0\\ • & • & -\rho \Omega_{55} \end{array}\right]\\ H_{23} & = & \left[\begin{array}{cccc} 0 & 0 & 0 & 0\\ 0 & 0 & 0 & 0\\ 0 & 0 & 0 & 0 \end{array}\right],\;{\hat{\Gamma }}_{1}\left[\begin{array}{c} \Gamma_{0}\\ 0 \end{array}\right]\\ H_{33} & = & \left[\begin{array}{cccc} -Z_{x} & 0 & 0 & 0\\ • & -Q_{x} & 0 & 0\\ • & • & -\Theta_{x} & 0\\ • & • & • & -\Theta_{x}^{t} \end{array}\right] \end{array}$$

$$\begin{array}{ccc} \Omega_{11} & = & \left[\begin{array}{cc} \Pi_{11} & B_{0}K_{I}+X_{x}+B_{0}Y\\ • & -N_{0}+4I \end{array}\right],\;\\ \;\Omega_{12} & = & \left[\begin{array}{cc} A_{do}-N_{1}-N_{2}+N_{3}+N_{4} & 0\\ 0 & 0 \end{array}\right]\\ \Omega_{13} & = & \left[\begin{array}{cc} 0 & 0\\ -I & 0 \end{array}\right],\;\;\Omega_{14}=\left[\begin{array}{cc} -\rho N_{1}-\rho N_{2} & 0\\ 0 & I \end{array}\right],\;\\ \;\Omega_{15} & = & \left[\begin{array}{cc} X_{x}A_{0}^{t}+B_{0}YA_{0}^{t}+W^{t}B_{0}^{t}+H^{t} & X_{x}+B_{0}Y\\ K_{I}^{t}B_{0}^{t} & 0 \end{array}\right]\\ \Omega_{16} & = & \left[\begin{array}{c} R_{x}\\ 0 \end{array}\right],\;\;\Omega_{17}=\left[\begin{array}{c} M_{x}\\ 0 \end{array}\right],\;\;\Omega_{18}=\left[\begin{array}{c} {𝒮}_{x}\\ 0 \end{array}\right]\;\;,\\ \Omega_{22} & = & \left[\begin{array}{cc} \Psi_{x}+\Psi_{x}^{t}+\left(1-\mu \right)Z_{x} & 0\\ 0 & (3-\mu )I \end{array}\right],\\ \Omega_{24} & = & \left[\begin{array}{cc} \Psi_{x} & 0\\ 0 & I \end{array}\right],\;\;\Omega_{25}=\left[\begin{array}{cc} A_{do}^{t} & 0\\ 0 & 0 \end{array}\right],\;\;\\ \Omega_{33} & = & \left[\begin{array}{cc} Q_{x} & 0\\ 0 & I \end{array}\right],\;\Omega_{35}=\left[\begin{array}{cc} 0 & -I\\ 0 & 0 \end{array}\right],\;\;\;\\ \;\Omega_{55} & = & \left[\begin{array}{cc} {\tilde{\Phi }}_{x} & 0\\ 0 & I \end{array}\right],\;\Omega_{44}=\left[\begin{array}{cc} M_{x} & 0\\ 0 & I \end{array}\right],\\ \Pi_{11} & = & A_{0}X_{x}+X_{x}A_{0}^{t}+B_{0}W+W^{t}B_{0}^{t}+A_{0}Y^{t}B_{0}^{t}\\ & & +B_{0}YA_{0}^{t}+H+H^{t},\;\\ {\tilde{\Phi }}_{x} & = & \left(I-B_{0}K_{D}\right)M_{x}^{-1}{\left(I-B_{0}K_{D}\right)}^{t} \end{array}$$

Furthermore, the controller parameters are obtained as $K_{P}={\mathrm{WX}}_{x}^{-1},$$K_{D}=-{\mathrm{YX}}_{x}^{-1}$ and $K_{I}$ is obtained directly from element (1,2) of $\Omega_{11}$.

**Proof.**  First, the stability of the considered system is proven. Consider the following LKF:

(27) $$\begin{array}{ccc} V_{2}\left(t\right) & = & V_{2a}\left(t\right)+V_{2b}\left(t\right)+V_{2c}\left(t\right)+V_{2d}\left(t\right)\\ V_{2a}\left(t\right) & = & \zeta^{t}\left(t\right)P\zeta \left(t\right),\;V_{2b}\left(t\right)=\int_{t-\tau (t)}^{t}\zeta^{t}\left(s\right)Z\zeta \left(s\right)ds\\ V_{2c}\left(t\right) & = & \int_{t-\rho }^{t}\zeta^{t}\left(s\right)Q\zeta \left(s\right)ds,\;\\ V_{2d}\left(t\right) & = & \int_{-\tau }^{t}\int_{t+\phi }^{t}\zeta^{t}\left(s\right)M\zeta \left(s\right)dsd\phi \\ M & = & \left[\begin{array}{cc} M_{x} & 0\\ 0 & I \end{array}\right],\Theta =\left[\begin{array}{cc} \Theta_{x} & 0\\ 0 & I \end{array}\right],\Psi =\left[\begin{array}{cc} \Psi_{x} & 0\\ 0 & I \end{array}\right] \end{array}$$

where P, Q and Z are as in (12). Using the Leibniz–Newton formula in (25), setting w(.)=0, evaluating ${\overset{\cdot }{V}}_{2}\left(t\right)$ along the solutions to (7) and after some algebraic manipulations, it is easy to show that ${\overset{\cdot }{V}}_{2}\left(t\right)$ can be written as

(28) $${\overset{\cdot }{V}}_{2}\left(t\right)=\frac{1}{\tau (t)}\int_{t-\tau (t)}^{t}X^{t}\left(t,\phi \right)\Xi_{s}X\left(t,\phi \right)d\phi$$

where

$$\begin{array}{ccc} & & X(t,\phi )=\\ & & \;{\left[\begin{array}{cccc} \zeta^{t}\left(t\right) & \zeta^{t}\left(t-\tau \left(t\right)\right) & \zeta^{t}\left(t-\rho \right) & \overset{\cdot }{\zeta^{t}}\left(\phi \right) \end{array}\right]}^{t} \end{array}$$

and

(29) $$\begin{array}{ccc} \Xi_{s} & = & \left[\begin{array}{cccc} \Xi_{as} & \Xi_{bs} & \Xi_{cs} & -\tau (t)\Theta \\ • & \Xi_{ds} & \Xi_{es} & -\tau (t)\Psi \\ • & • & \Xi_{fs} & 0\\ • & • & • & -\tau (t)M \end{array}\right]\;\\ \Xi_{as} & = & PA_{a}+A_{a}P+\Theta +\Theta^{t}+Z+Q+\tau \left(t\right)A_{a}^{t}{\mathrm{MA}}_{a}\\ \Xi_{bs} & = & PA_{do}-\Theta +\Psi^{t}+\tau \left(t\right)A_{a}^{t}{\mathrm{MA}}_{do}\\ \Xi_{cs} & = & PA_{c}+\tau \left(t\right)A_{a}^{t}{\mathrm{MA}}_{c}\\ \Xi_{ds} & = & -\Psi -\Psi^{t}-\left(1-\mu \right)Z+\tau \left(t\right)A_{do}^{t}{\mathrm{MA}}_{d}\\ \Xi_{es} & = & \tau \left(t\right)A_{do}^{t}{\mathrm{MA}}_{c}\\ \Xi_{fs} & = & -Q+\tau \left(t\right)A_{c}^{t}{\mathrm{MA}}_{c} \end{array}$$

The relaxation matrices Θ and Ψ are introduced to facilitate the delay-dependent analysis. When $\Xi_{s}<0,$ then ${\overset{\cdot }{V}}_{2}\left(t\right)<0$ for any X(t,ϕ)≠0 and all τ≤ρ.

Let us write (29) as

(30) $$\begin{array}{ccc} \Xi_{s} & = & \\ & & \;\left[\begin{array}{cccc} {\hat{\Xi }}_{as} & PA_{do}-\Theta +\Psi^{t} & PA_{c} & -\tau (t)\Theta \\ • & -\Psi -\Psi^{t}-\left(1-\mu \right)Z & 0 & -\tau (t)\Psi \\ • & • & -Q & 0\\ • & • & • & -\tau (t)M \end{array}\right]\\ & & \;+\left[\begin{array}{cccc} \tau \left(t\right)A_{a}^{t}{\mathrm{MA}}_{a} & \tau \left(t\right)A_{a}^{t}{\mathrm{MA}}_{do} & \tau \left(t\right)A_{a}^{t}{\mathrm{MA}}_{c} & 0\\ • & \tau \left(t\right)A_{do}^{t}{\mathrm{MA}}_{do} & \tau \left(t\right)A_{do}^{t}{\mathrm{MA}}_{c} & 0\\ • & • & \tau \left(t\right)A_{c}^{t}{\mathrm{MA}}_{c} & 0\\ • & • & 0 & 0 \end{array}\right] \end{array}$$

where

$${\hat{\Xi }}_{as}=PA_{a}+A_{a}P+\Theta +\Theta^{t}+Z+Q$$

Using Schur’s complement, (30) can be written for all 0<τ(t)<ρ as

(31) $$\begin{array}{ccc} \Xi_{s_{1}} & = & \\ & & \;\left[\begin{array}{ccccc} {\hat{\Xi }}_{as} & {\mathrm{PA}}_{do}-\Theta +\Psi^{t} & {\mathrm{PA}}_{c} & -\tau (t)\Theta & \tau \left(t\right)A_{a}^{t}P\\ • & -\Psi -\Psi^{t}-\left(1-\mu \right)Z & 0 & -\tau (t)\Psi & \tau \left(t\right)A_{do}^{t}P\\ • & • & -Q & 0 & \tau \left(t\right)A_{c}^{t}P\\ • & • & • & -\tau (t)M & 0\\ • & • & • & • & -\tau (t)\Phi \end{array}\right] \end{array}$$

Substituting the upper bound of τ(t) into (31), we obtain

(32) $$\begin{array}{ccc} \Xi_{s_{2}} & = & \\ & & \;\left[\begin{array}{ccccc} {\hat{\Xi }}_{as} & {\mathrm{PA}}_{do}-\Theta +\Psi^{t} & {\mathrm{PA}}_{c} & -\rho \Theta & \rho A_{a}^{t}P\\ • & -\Psi -\Psi^{t}-\left(1-\mu \right)Z & 0 & -\rho \Psi & \rho A_{do}^{t}P\\ • & • & -Q & 0 & \rho A_{c}^{t}P\\ • & • & • & -\rho M & 0\\ • & • & • & • & -\rho \Phi \end{array}\right] \end{array}$$

where $\Phi ={\mathrm{PM}}^{-1}P.$ For ${\overset{\cdot }{V}}_{2}\left(t\right)$ to be less than zero, $\Xi_{s_{2}}$ in (32) should be less than zero. By applying the congruent transformation

$$diag\left[\begin{array}{ccccc} T_{1} & I & I & I & T_{1} \end{array}\right],$$

to (32), expanding its elements and simplifying, the following inequality is obtained.

(33) $$\left[\begin{array}{ccccc} U_{11} & U_{12} & U_{13} & U_{14} & U_{15}\\ {}* & U_{22} & U_{23} & U_{24} & U_{25}\\ {}* & * & U_{33} & U_{34} & U_{35}\\ {}* & * & * & U_{44} & U_{45}\\ {}* & * & * & * & U_{55} \end{array}\right]<0$$

where

$$\begin{array}{ccc} U_{11} & = & \left[\begin{array}{cc} \Pi_{11}+{\hat{\Pi }}_{12} & B_{0}K_{I}+X_{x}+B_{0}Y\\ • & -N_{0}+4I \end{array}\right]\\ U_{12} & = & \left[\begin{array}{cc} A_{do}-N_{1}-N_{2}+N_{3}+N_{4} & 0\\ 0 & 0 \end{array}\right]\\ U_{13} & = & \left[\begin{array}{cc} 0 & 0\\ -I & 0 \end{array}\right],\;U_{14}=\rho \left[\begin{array}{cc} -N_{1}-N_{2} & 0\\ 0 & I \end{array}\right]\\ U_{15} & = & \rho \left[\begin{array}{cc} X_{x}A_{0}^{t}+B_{0}YA_{0}^{t}+W^{t}B_{0}^{t}+H^{t} & X_{x}+B_{0}Y\\ K_{I}^{t}B_{0}^{t} & 0 \end{array}\right] \end{array}$$

$$\begin{array}{ccc} U_{22} & = & -\left[\begin{array}{cc} \Psi_{x}+\Psi_{x}^{t}+\left(1-\mu \right)Z_{x} & 0\\ 0 & (3-\mu )I \end{array}\right]\\ U_{23} & = & \left[\begin{array}{cc} 0 & 0\\ 0 & 0 \end{array}\right],\;U_{24}=-\rho \left[\begin{array}{cc} \Psi_{x} & 0\\ 0 & I \end{array}\right]\\ U_{25} & = & \rho \left[\begin{array}{cc} A_{do}^{t} & 0\\ 0 & 0 \end{array}\right],\;U_{33}=-\left[\begin{array}{cc} Q_{x} & 0\\ 0 & I \end{array}\right]\\ U_{34} & = & \left[\begin{array}{cc} 0 & 0\\ 0 & 0 \end{array}\right],\;U_{35}=\rho \left[\begin{array}{cc} 0 & -I\\ 0 & 0 \end{array}\right]\\ U_{44} & = & -\rho \left[\begin{array}{cc} M_{x} & 0\\ 0 & I \end{array}\right],\;U_{45}=\left[\begin{array}{cc} 0 & 0\\ 0 & 0 \end{array}\right]\\ U_{55} & = & -\rho \left[\begin{array}{cc} {\tilde{\Phi }}_{x} & 0\\ 0 & I \end{array}\right] \end{array}$$

$$\begin{array}{ccc} \Pi_{11} & = & A_{0}X_{x}+X_{x}A_{0}^{t}+B_{0}W+W^{t}B_{0}^{t}+A_{0}Y^{t}B_{0}^{t}\\ & & +B_{0}YA_{0}^{t}+H+H^{t}\\ {\hat{\Pi }}_{12} & = & \left(X_{x}+B_{0}Y\right)Z_{x}{\left(X_{x}+B_{0}Y\right)}^{t}\\ & & +\left(X_{x}+B_{0}Y\right)Q_{x}{\left(X_{x}+B_{0}Y\right)}^{t}\\ & & +T_{1}\Theta T_{1}^{t}+T_{1}\Theta^{t}T_{1}^{t}\\ & = & \left(X_{x}+B_{0}Y\right)Z_{x}Z_{x}^{-1}Z_{x}{\left(X_{x}+B_{0}Y\right)}^{t}\\ & & +\left(X_{x}+B_{0}Y\right)Q_{x}Q_{x}^{-1}Q_{x}{\left(X_{x}+B_{0}Y\right)}^{t}\\ & & +𝒮\Theta_{x}^{-1}{𝒮}^{t}+𝒮\Theta_{x}^{-t}{𝒮}^{t}\\ & = & {\mathrm{RZ}}_{x}^{-1}R^{t}+{\mathrm{MQ}}_{x}^{-1}M^{t}+𝒮\Theta_{x}^{-1}{𝒮}^{t}+𝒮\Theta_{x}^{-t}{𝒮}^{t}\\ H & = & B_{0}K_{P}X_{x}K_{D}^{t}B_{0}^{t},\;\;\;R_{x}=\left(X_{x}+B_{0}Y\right)Z_{x},\;\;\;\\ L_{x} & = & \left(X_{x}+B_{0}Y\right)Q_{x},\;{𝒮}_{x}=\left(I-B_{0}K_{D}\right)X_{x}\Theta_{x} \end{array}$$

which may be written in a short notation as

(34) $${\tilde{\Xi }}_{s}=\left[\begin{array}{ccccc} {\hat{\Omega }}_{11} & \Omega_{12} & \Omega_{13} & \Omega_{14} & \rho \Omega_{15}\\ • & -\Omega_{22} & 0 & -\rho \Omega_{24} & \rho \Omega_{25}\\ • & • & -\Omega_{33} & 0 & \rho \Omega_{35}\\ • & • & • & -\rho \Omega_{44} & 0\\ • & • & • & • & -\rho \Omega_{55} \end{array}\right]<0$$

where

$${\hat{\Omega }}_{11}=\Omega_{11}+\left[\begin{array}{cc} {\hat{\Pi }}_{12} & 0\\ 0 & 0 \end{array}\right],\;\;$$

and the rest of $\Omega^{{}^{\prime }s}$ are defined in (26). Using Schur’s complement on the second term of ${\hat{\Omega }}_{11}$, we obtain

(35) $$\left[\begin{array}{cc} \Upsilon_{11} & \Upsilon_{12}\\ {}* & \Upsilon_{22} \end{array}\right]<0$$

where

$$\begin{array}{ccc} \Upsilon_{11} & = & \left[\begin{array}{ccccc} \Omega_{11} & \Omega_{12} & \Omega_{13} & \Omega_{14} & \rho \Omega_{15}\\ • & -\Omega_{22} & 0 & -\rho \Omega_{24} & \rho \Omega_{25}\\ • & • & -\Omega_{33} & 0 & \rho \Omega_{35}\\ • & • & • & -\rho \Omega_{44} & 0\\ • & • & • & • & -\rho \Omega_{55} \end{array}\right]\\ \Upsilon_{12} & = & \left[\begin{array}{cccc} \Omega_{16} & \Omega_{17} & \Omega_{18} & \Omega_{18}\\ 0 & 0 & 0 & 0\\ 0 & 0 & 0 & 0\\ 0 & 0 & 0 & 0\\ 0 & 0 & 0 & 0 \end{array}\right]\\ \Upsilon_{22} & = & \left[\begin{array}{cccc} -Z_{x} & 0 & 0 & 0\\ • & -Q_{x} & 0 & 0\\ • & • & -\Theta_{x} & 0\\ • & • & • & -\Theta_{x}^{t} \end{array}\right] \end{array}$$

From (35), the asymptotic stability of the closed-loop systems (7) and (8) are established since ${\overset{\cdot }{V}}_{2}\left(t\right)<0$ in (28).

Next, consider the performance measure

(36) $$J=\int_{0}^{\infty }\left[z^{t}\left(s\right)z\left(s\right)-\gamma^{2}w^{t}\left(s\right)w\left(s\right)\right]ds$$

For any $w\left(t\right)\in L_{2}\left(0,\infty \right)\neq 0$ and zero initial condition x(0)=0, we have

(37) $$\begin{array}{ccc} J & = & \int_{0}^{\infty }\left[z{\left(s\right)}^{t}z\left(s\right)-\gamma^{2}w^{t}\left(s\right)w\left(s\right)+{\left.{\overset{\cdot }{V}}_{2}\left(t\right)\right|}_{\left(7\right)}\right]ds\\ & & -{\left.{\overset{\cdot }{V}}_{2}\left(t\right)\right|}_{\left(7\right)}\\ & \leq & \int_{0}^{\infty }\left[z{\left(s\right)}^{t}z\left(s\right)-\gamma^{2}w^{t}\left(s\right)w\left(s\right)+{\left.{\overset{\cdot }{V}}_{1}\left(t\right)\right|}_{\left(7\right)}\right]ds \end{array}$$

Proceed to compute the expression

$$z^{t}\left(s\right)z\left(s\right)-\gamma^{2}\omega^{t}\left(s\right)\omega \left(s\right)+\overset{\cdot }{V}\left(s\right)=\tilde{\eta }\left(s\right)\Xi_{s}^{t}\tilde{\eta }\left(s\right)$$

First, from (18) we can obtain the expression

$$z^{t}\left(s\right)z\left(s\right)-\gamma^{2}\omega^{t}\left(s\right)\omega \left(s\right)={\tilde{\eta }}^{t}\left(s\right){\bar{\Sigma }}_{\infty }\tilde{\eta }\left(s\right)$$

where

$${\bar{\Sigma }}_{\infty }=\left[\begin{array}{ccccc} {\hat{G}}_{o}^{t}{\hat{G}}_{o} & {\hat{G}}_{o}^{t}{\hat{G}}_{do} & 0\; & 0 & {\hat{G}}_{o}^{t}\Phi_{o}\\ {\hat{G}}_{do}^{t}{\hat{G}}_{o} & {\hat{G}}_{do}^{t}{\hat{G}}_{do} & 0 & 0 & {\hat{G}}_{do}^{t}\Phi_{o}\\ 0 & 0 & 0 & 0 & 0\\ 0 & 0 & 0 & 0 & 0\\ \Phi_{o}^{t}{\hat{G}}_{o} & \Phi_{o}^{t}{\hat{G}}_{do} & 0 & 0 & -\gamma^{2}I+\Phi_{o}^{t}\Phi_{o} \end{array}\right]$$

and

$$\tilde{\eta }\left(s\right)={\left[\begin{array}{ccccc} \zeta^{t}\left(s\right) & \zeta^{t}\left(s-\tau \right) & \zeta^{t}\left(s-\rho \right) & \overset{\cdot }{\zeta^{t}}\left(\phi \right) & \omega^{t}\left(s\right) \end{array}\right]}^{t}$$

Accordingly, (29) is expanded and modified into

(38) $${\bar{\Xi }}_{s}=\left[\begin{array}{ccccc} \Xi_{as} & \Xi_{bs} & \Xi_{cs} & -\tau (t)\Theta & P{\hat{\Gamma }}_{1}\\ • & \Xi_{ds} & \Xi_{es} & -\tau (t)\Psi & 0\\ • & • & \Xi_{fs} & 0 & 0\\ • & • & • & -\tau (t)M & 0\\ • & • & • & • & 0 \end{array}\right]$$

Separating (38) as was performed in (30), then using Schur’s complement and applying the congruent transformation $diag\left[\begin{array}{cccccc} T_{1} & I & I & I & I & T_{1} \end{array}\right]$ to ${\bar{\Xi }}_{s},$ with some algebraic manipulations, (38) becomes

$${\bar{\Xi }}_{s}=\left[\begin{array}{cccccc} {\hat{\Omega }}_{11} & \Omega_{12} & \Omega_{13} & \Omega_{14} & {\hat{\Gamma }}_{1} & \rho \Omega_{15}\\ • & -\Omega_{22} & 0 & -\rho \Omega_{24} & 0 & \rho \Omega_{25}\\ • & • & -\Omega_{33} & 0 & 0 & \rho \Omega_{35}\\ • & • & • & -\rho \Omega_{44} & 0 & 0\\ • & • & • & • & 0 & 0\\ • & • & • & • & • & -\rho \Omega_{55} \end{array}\right]$$

Incorporating ${\bar{\Sigma }}_{\infty }$ and ${\bar{\Xi }}_{s},$

$$\begin{array}{ccc} {\tilde{\Xi }}_{s} & = & \left[\begin{array}{ccc} {\hat{\Omega }}_{11}+{\hat{G}}_{o}^{t}{\hat{G}}_{o} & \Omega_{12}+{\hat{G}}_{o}^{t}{\hat{G}}_{do} & \Omega_{13}\\ • & -\Omega_{22}+{\hat{G}}_{do}^{t}{\hat{G}}_{do} & 0\\ • & • & -\Omega_{33}\\ • & • & •\\ • & • & •\\ • & • & • \end{array}\right.\\ & & \left.\begin{array}{ccc} \Omega_{14} & {\hat{\Gamma }}_{0}+{\hat{G}}_{o}^{t}\Phi_{o} & \rho \Omega_{15}\\ -\rho \Omega_{24} & {\hat{G}}_{do}^{t}\Phi_{o} & \rho \Omega_{25}\\ 0 & 0 & \rho \Omega_{35}\\ -\rho \Omega_{44} & 0 & 0\\ • & -\gamma^{2}I+\Phi_{o}^{t}\Phi_{o} & 0\\ • & • & -\rho \Omega_{55} \end{array}\right] \end{array}$$

The term ${\hat{\Omega }}_{11}$ includes ${\hat{\Pi }}_{12}$, defined in (33) as

$${\hat{\Pi }}_{12}={\mathrm{RZ}}_{x}^{-1}R^{t}+{\mathrm{MQ}}_{x}^{-1}M^{t}+𝒮\Theta_{x}^{-1}{𝒮}^{t}+𝒮\Theta_{x}^{-t}{𝒮}^{t}$$

Using Schur’s complement for this term, we obtain

$$\begin{array}{ccc} {\tilde{\Xi }}_{s} & = & \left[\begin{array}{ccc} \Omega_{11}+{\hat{G}}_{o}^{t}{\hat{G}}_{o} & \Omega_{12}+{\hat{G}}_{o}^{t}{\hat{G}}_{do} & \Omega_{13}\\ • & -\Omega_{22}+{\hat{G}}_{do}^{t}{\hat{G}}_{do} & 0\\ • & • & -\Omega_{33}\\ • & • & •\\ • & • & •\\ • & • & •\\ • & • & •\\ • & • & •\\ • & • & •\\ • & • & • \end{array}\right.\\ & & \left.\begin{array}{ccc} \Omega_{14} & {\hat{\Gamma }}_{1}+{\hat{G}}_{o}^{t}\Phi_{o} & \rho \Omega_{15}\\ -\rho \Omega_{24} & {\hat{G}}_{do}^{t}\Phi_{o} & \rho \Omega_{25}+\\ 0 & 0 & \rho \Omega_{35}\\ -\rho \Omega_{44} & 0 & 0\\ • & -\gamma^{2}I+\Phi_{o}^{t}\Phi_{o} & 0\\ • & • & -\rho \Omega_{55}\\ • & • & •\\ • & • & •\\ • & • & •\\ • & • & • \end{array}\right.\\ & & \left.\begin{array}{cccc} \Omega_{16} & \Omega_{167} & \Omega_{18} & \Omega_{18}\\ 0 & 0 & 0 & 0\\ 0 & 0 & 0 & 0\\ 0 & 0 & 0 & 0\\ 0 & 0 & 0 & 0\\ 0 & 0 & 0 & 0\\ -Z_{x} & 0 & 0 & 0\\ • & -Q_{x} & 0 & 0\\ • & • & -\Theta_{x} & 0\\ • & • & • & -\Theta_{x}^{t} \end{array}\right] \end{array}$$

Therefore, (26) is obtained. □

## 4. Simulation

In this section, we demonstrate the application of the foregoing analytical results on two operating points of a typical system. The results of Theorem 2 are reported here. Implementation of the developed theorems was accomplished using the MATLAB LMI-solver. The LIM-solver was used to find the unknown quantities in LMI (26). Then the PID parameters were calculated as stated in the theorem.

Model 1:

$$\begin{array}{ccc} A_{o} & = & \left[\begin{array}{cccc} -2.1 & 1 & 1 & 1\\ 3 & 0 & 0 & 2\\ -1 & 0 & -1.9 & -3\\ -2 & -1 & 2 & -1.1 \end{array}\right],\;\;\;\;\\ A_{do} & = & \left[\begin{array}{cccc} -0.3 & 0 & 0.6 & 0\\ 0 & -1 & 0 & -0.8\\ 0 & -0.8 & 0 & -1.3\\ 0.1 & 0 & 0.5 & 0 \end{array}\right]\\ \Gamma_{o} & = & \left[\begin{array}{cccc} 1 & 0 & 0 & 0\\ 0 & 0 & 0 & 0\\ 1 & 0 & 0 & 0\\ 0 & 0 & 0 & 0 \end{array}\right],\;\;\\ \;\Phi_{o} & = & \left[\begin{array}{cccc} 0.1 & 0 & 0 & 0\\ 0 & 0.3 & 0 & 0\\ 0 & 0 & 0.2 & 0\\ 0 & 0 & 0 & 0 \end{array}\right]\\ B_{o} & = & \left[\begin{array}{cc} 0 & 0\\ 1 & 0\\ 0 & 0\\ 0 & 1 \end{array}\right],\;\;\;G_{o}=\left[\begin{array}{cccc} 1 & 0 & -1 & 0\\ 0 & 0 & 0 & 0\\ 0 & 0 & 0 & 0\\ 0 & 0 & 0 & 0 \end{array}\right],\;\;\;\\ G_{do} & = & \left[\begin{array}{cccc} 0.1 & 0 & 0.1 & 0\\ 0 & 0 & 0 & 0\\ 0 & 0 & 0 & 0\\ 0 & 0 & 0 & 0 \end{array}\right] \end{array}$$

The time-delay pattern caused by the DoS attack is τ(t)=0.1cos(2πft), where f=0.75 Hz. The simulation parameters for operating point 1: ρ=0.1 s, μ=0.5, and γ=0.35. The PID-like controller’s gains for operating point 1 are as follows:

$$\begin{array}{ccc} K_{P} & = & \left[\begin{array}{cccc} -3.2036 & -1.3251 & -0.4306 & -1.1852\\ 0.7998 & -0.6954 & -1.3093 & 0.2105 \end{array}\right]\\ K_{D} & = & \left[\begin{array}{cccc} -0.1673 & -0.5084 & 1.1232 & -0.5361\\ -0.2389 & -1.0250 & -2.3911 & 0.4363 \end{array}\right]\\ K_{I} & = & \left[\begin{array}{cccc} 0.1894 & -1.0705 & -2.7097 & 0.0442\\ 2.9293 & 1.8180 & 4.5353 & -3.0460 \end{array}\right] \end{array}$$

Model 2:

$$\begin{array}{ccc} A_{o} & = & \left[\begin{array}{cccc} -2.2 & 1 & 1 & 1\\ 3 & 0 & 0 & 2\\ -1 & 0 & -2.1 & -3\\ -2 & -1.1 & 2 & -1 \end{array}\right],\;\;\;\;\\ A_{do} & = & \left[\begin{array}{cccc} -0.2 & 0 & 0.6 & 0\\ 0 & -1 & 0 & -0.7\\ 0 & -0.9 & 0 & -1.2\\ 0.1 & 0 & 0.5 & 0 \end{array}\right]\\ \Gamma_{o} & = & \left[\begin{array}{cccc} 1 & 0 & 0 & 0\\ 0 & 0 & 0 & 0\\ 1 & 0 & 0 & 0\\ 0 & 0 & 0 & 0 \end{array}\right],\;\;\;\\ \Phi_{o} & = & \left[\begin{array}{cccc} 0.2 & 0 & 0 & 0\\ 0 & 0.1 & 0 & 0\\ 0 & 0 & 0.3 & 0\\ 0 & 0 & 0 & 0 \end{array}\right]\\ B_{o} & = & \left[\begin{array}{cc} 0 & 0\\ 1 & 0\\ 0 & 0\\ 0 & 1 \end{array}\right],\;\;\;G_{o}=\left[\begin{array}{cccc} 0 & 1 & 0 & -1\\ 0 & 0 & 0 & 0\\ 0 & 0 & 0 & 0\\ 0 & 0 & 0 & 0 \end{array}\right],\;\;\\ \;G_{do} & = & \left[\begin{array}{cccc} 0.2 & 0 & 0.2 & 0\\ 0 & 0 & 0 & 0\\ 0 & 0 & 0 & 0\\ 0 & 0 & 0 & 0 \end{array}\right] \end{array}$$

The simulation parameters for operating point 2: ρ=0.1 s, μ=0.5, and γ=0.3. The PID-like controller’s gains for operating point 2 are as follows:

$$\begin{array}{ccc} K_{P} & = & \left[\begin{array}{cccc} -3.1188 & -2.1009 & -1.0002 & 0.0610\\ 1.4442 & 0.4500 & -1.6441 & -0.9490 \end{array}\right]\\ K_{D} & = & \left[\begin{array}{cccc} -7.0332 & -19.5765 & -6.0811 & 18.9382\\ 16.0464 & -29.9312 & 21.5104 & -21.7494 \end{array}\right]\\ K_{I} & = & \left[\begin{array}{cccc} 0.2035 & -0.7838 & 0.1681 & -0.1392\\ -0.1583 & 0.2414 & -0.4235 & -0.7837 \end{array}\right] \end{array}$$

**Figure 1:**
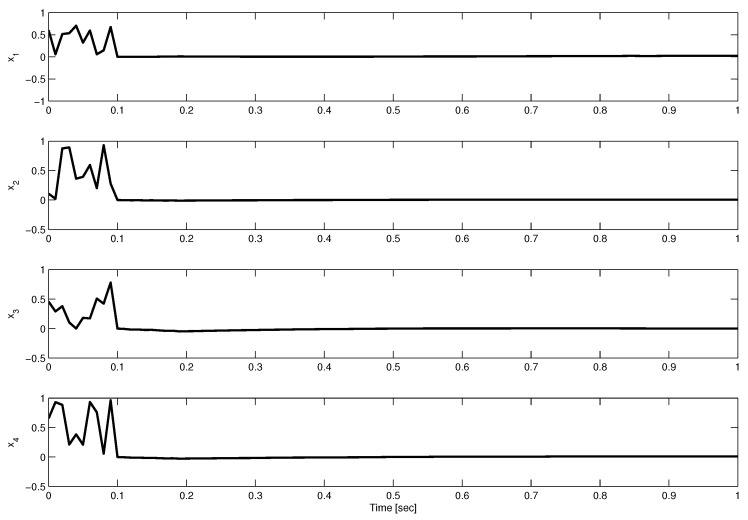
Evolution of state trajectories at operating point 1.

**Figure 2:**
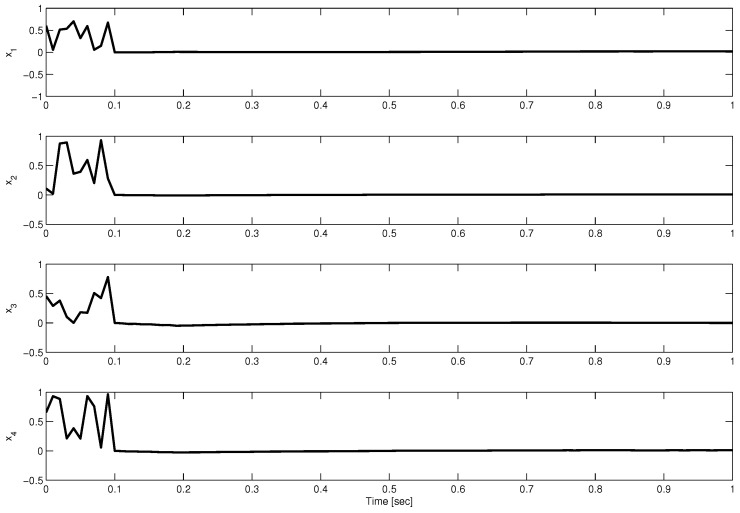
Evolution of state trajectories at operating point 2.

From the time-delay pattern caused by the DoS attacks, the maximum bounds, ρ and μ, can easily be verified. For both operating points, random noises with a maximum magnitude of 0.1 are taken as disturbances. The initial values of the states are also taken as random numbers between 0 and 1. Figure 1 and Figure 2 show the state trajectories under the proposed controller.

The obtained results show that the proposed strategy yield less performance bound γ, thus providing improved stabilization for the combustion in the rocket motor chambers model with two operating modes.

## 5. Conclusions

This paper presented a new approach for stabilizing a class of linear time-delay systems that are subject to DoS attacks. The method takes into account the different ways that a DoS attack can impact the system, specifically its delay-independent and -dependent behaviour. To overcome these behaviours, the authors employed an LKF and derive new linear matrix inequalities. They demonstrated the effectiveness of their method through numerical examples for both delay-independent and -dependent robust stabilization. In general, the results of this study are expected to be useful for understanding and controlling NCSs in the presence of DoS attacks. An extension of the presented results could be to include state observers to estimate the states when they are not measurable. Furthermore, a mathematical model for the cyber-attack may be obtained and augmented with the time-delay system.

## Data Availability

The data generated during the current study are available from the corresponding author upon reasonable request.
